# Photoactivation‐Enabled Tunable Room‐Temperature Phosphorescence and Thermally Activated Delayed Fluorescence in Crystalline Materials

**DOI:** 10.1002/advs.77468

**Published:** 2026-08-29

**Authors:** Lisha Zhang, Yongping Wu, Ihor Sahalianov, Lei Zhou, Glib Baryshnikov, Xiang Ma

**Affiliations:** ^1^ Key Laboratory for Advanced Materials and Feringa Nobel Prize Scientist Joint Research Center Frontiers Science Center for Materiobiology and Dynamic Chemistry School of Chemistry and Molecular Engineering East China University of Science and Technology Shanghai China; ^2^ Laboratory of Organic Electronics Department of Science and Technology Linköping University Norrköping Sweden

**Keywords:** crystalline material, photoactivation, room‐temperature phosphorescence, thermally activated delayed fluorescence

## Abstract

Photoactivation‐enabled tunable room‐temperature phosphorescence (RTP) and thermally activated delayed fluorescence (TADF) are rarely realized in crystalline materials because of their rigid molecular packing. Existing crystalline photoactivated RTP systems generally require pretreatment and have not enabled RTP/TADF regulation. Here, we demonstrate for the first time that intrinsic photoactivated RTP can be achieved in a pretreatment‐free crystalline system, while the occurrence of TADF can be switched on or off through rational host‐guest engineering. Dinaphtho[2,1‐b:1′,2′‐d]furan (OA) exhibits intrinsic photoactivated RTP at 77 K, when incorporated into three crystalline hosts, forms a general crystalline photoactivated platform at room temperature. Experimental and calculational results indicate that energy level matching and exciton lifetime govern RTP/TADF modulation. Specifically, long‐lived excitons in host dibenzofuran (DBF) enable simultaneous RTP and TADF through host‐guest energy transfer, whereas methyl 4‐(methylamino)benzoate (MAB) and boric acid (BA) as hosts suppress TADF due to mismatched energy levels or rapid exciton quenching. Nevertheless, their RTP emission is facilitated by high crystallinity and rigid hydrogen‐bonding networks. Mechanistic analysis indicates that UV irradiation regulates excited‐state carrier concentration and transition probability, thereby enabling photoactivation. This work establishes a design strategy for crystalline photoactivated luminescent materials with potential applications for time‐dependent anti‐counterfeiting.

## Graphical abstract

This work is the first to demonstrate the regulation of photoactivated room‐temperature phosphorescence (RTP) and thermally activated delayed fluorescence (TADF) in crystalline materials without any pretreatment. Furthermore, we reveal the underlying mechanisms by uncovering the role of host‐guest energy level alignment, exciton lifetime, crystallinity, excited‐state carrier concentrations, and transition probabilities in governing the luminescent behavior.

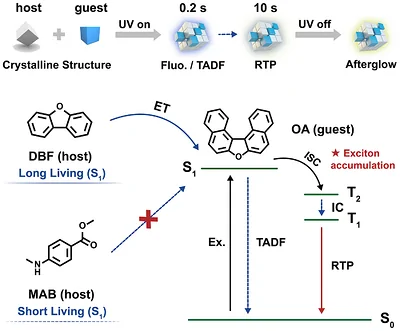

## 1 Introduction

Dynamic modulation of luminescence in crystalline materials [1, 2], specifically, in‐situ reversible switching between room‐temperature phosphorescence (RTP) and thermally activated delayed fluorescence (TADF) via photoactivation, matters for next‐generation optoelectronics and information encryption. In amorphous polymer matrices like polymethyl methacrylate (PMMA), the loose structure permits triplet oxygen consumption by RTP dopants [3], which drives a typical oxygen‐depletion photoactivation process. Crystalline materials, by contrast, pack molecules tightly with very low oxygen permeability, so the oxygen‐depletion mechanism barely works. Meanwhile, the photo‐induced structural changes of the molecules require more specific conditions. For instance, tetraphenylethylene (TPE) undergoes an intramolecular photocyclodehydrogenation reaction under UV irradiation [4, 5]. Such photochemical reactions generally serve as an effective strategy for fluorescence modulation. For these reasons, photoactivation‐enabled tunable emission has long seemed out of reach in crystalline systems.

Progress in amorphous‐based photoactivated afterglow materials is ongoing. In 2017, Adachi et al. [6] reported an organic host‐guest system where long‐lived charge‐separated states recombined to produce exciplex emission, lasting over one hour at room temperature after photoactivation. In 2024, Chi et al. [7] designed a universal molecular host (DPOBP‐Br) and doped it with commercial fluorescent dyes, obtaining green‐to‐red dynamic RTP emission modulated by residual oxygen concentration in the amorphous matrix. These systems perform well, but amorphous matrices have inherent drawbacks: low thermal stability, hygroscopicity, and inconsistent oxygen permeability across batches [8]. These issues make it hard to meet reliability requirements in real applications like anti‐counterfeiting and photonic logic gates. Crystalline photoactivated materials, meanwhile, remain rare. In 2024, Li et al. [9] reported the first grinding‐induced photoactivated crystalline RTP material, in which UV irradiation initiated the formation of more folded molecular conformations and interlocked π–π stacking that stabilized triplet excitons. However, the photoactivation required specific grinding pretreatment and produced only RTP, without TADF or dynamic switching. Developing a material system that enables intrinsic, pretreatment‐free photoactivation and dynamic tunability between RTP and TADF in the crystalline state remains an open problem.

**FIGURE 1:**
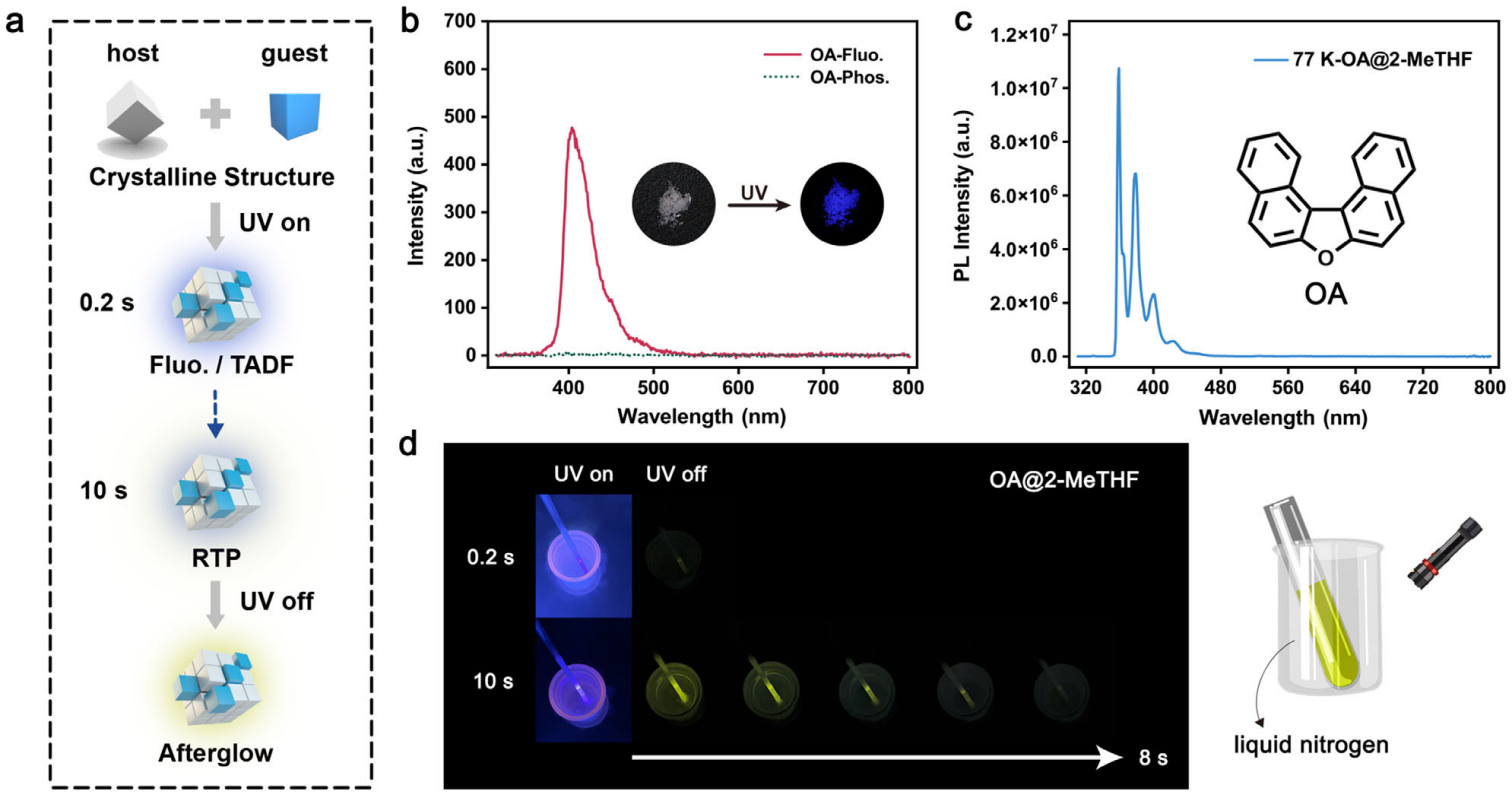
(a) The schematic diagram of the time‐dependent luminescence evolution in a host‐guest crystalline system upon UV irradiation. (b) The fluorescent and phosphorescent spectra of OA powder at room temperature. (c) The PL spectrum of OA in 2‐MeTHF at 77 K (c = 3.0 × 10^−5^ mol·L^−1^). The molecular formula of OA. (d) Time‐dependent afterglow photographs of OA@2‐MeTHF at 77 K. Experimental schematic of a liquid‐nitrogen‐cooled quartz electron paramagnetic resonance (EPR) tube.

To tackle these existing limitations, herein, we found that dinaphtho[2,1‐b1′,2′‐d]furan (OA) exhibited pronounced photoactivation in dilute 2‐methyltetrahydrofuran (2‐MeTHF) solution at 77 K. Inspired by the isomer‐design and doping strategy reported by Liu et al. [10] in 2022, we first employed dibenzofuran (DBF), a structural analogue of OA, as the host matrix to construct a doped RTP system. Guided by energy level matching, we subsequently expanded the host scope to additional crystalline matrices, methyl 4‐(methylamino)benzoate (MAB) and boric acid (BA). This stepwise host selection enabled the construction of a general crystalline photoactivated RTP system through simple room‐temperature doping (Figure 1a). The three hosts possess distinct photophysical properties, yet they all enhance the phosphorescence of OA by providing rigid microenvironments that suppress non‐radiative decay. Energy level matching and exciton lifetime have proven to be central to the simultaneous generation of RTP and TADF in OA‐doped systems. When energy levels of host and guest align appropriately, differences in host crystallinity can further modulate the emission behavior. Mechanistic investigations reveal that photoactivation originates from UV‐induced changes in excited‐state carrier population and radiative transition probability, leading to enhanced emissive processes. These results establish a guideline for designing crystalline photoactivated phosphorescent materials, with practical applicability in time‐dependent information encryption.

## 2 Results and Discussion

After purification (see Experimental Section 4.3 for details), the molecular structure of OA was characterized by using ^1^H NMR (400 MHz, DMSO‐*d*_6_), ^13^C NMR (101 MHz, DMSO‐*d*_6_), and high‐resolution electron impact (EI) mass spectrometry (Figures S1–S3). Experimental results also demonstrated that OA is a chromophore with high photoluminescence quantum yield (PLQY), reaching up to 80% (Figure S4). As shown in Figure 1b, OA only exhibited fluorescent emission without any detectable phosphorescence at room temperature. The photoluminescence (PL) spectrum of its dilute solution in 2‐MeTHF at 77 K is given in Figure 1c, where the emission peaks were primarily located in the violet region, consisting of several narrow bands. Under frozen conditions at 77 K, OA@2‐MeTHF exhibited significant photoactivated phosphorescent characteristics (Figure 1d). Specifically, when briefly exposed to UV light, OA@2‐MeTHF emitted a weak yellow afterglow for about 0.5 s. Moreover, after continuous UV irradiation, it emitted a bright yellow afterglow with a duration of up to 8 s.

The absorption and PL spectra of OA dissolved in different polarity solvents (hexane, toluene, tetrahydrofuran (THF), acetone, N,N‐dimethylformamide (DMF)) are shown in Figure S5 (c = 3.0 × 10^−5^ mol·L^−1^). OA exhibited multiple absorption peaks in all solvents, especially from 250 nm to 400 nm. Notably, the peak positions were nearly unchanged across different solvents, indicating that the ground‐state electronic structure is insensitive to solvent polarity due to the rigid and extended π‐conjugated framework [11]. The emission peaks were mainly located between 360 nm and 400 nm, displaying a slight red shift with increased solvent polarity, which might be attributed to the conjugated system and polar excited state of OA. The intramolecular charge transfer (ICT) [12] characteristic is very weak.

To achieve photoactivated RTP, we screened three crystalline host matrices, named DBF, MAB, and BA. The commercial starting materials were purified through silica gel column chromatography and recrystallization methods; their molecular structures are characterized in Figures S6–S11. The specific preparation methods for the doped materials are described in Experimental Section 4.4. For OA@DBF, we investigated the effect of doping concentration on phosphorescent performance. Figure S12 shows the delayed emission lifetime of samples with different doping concentrations (0.01 wt%, 0.1 wt%, 1 wt%, and 10 wt%) under the same measurement parameters. Among them, the one with 1 wt% doping concentration exhibited the longest lifetime (∼ 551.82 ms) under in‐situ photoactivation. As can be seen, when the doping concentration was too low, OA@DBF exhibited a short lifetime due to the lack of enough luminescent centers. Furthermore, higher doping concentration had a negligible effect in this regard, implying that below a concentration of 10 wt%, the quenching effect caused by molecular aggregation could be ignored. The doping concentration of 1 wt% ensures sufficient luminescent center density and exhibits optimal luminescent performance, thus being finally chosen.

**FIGURE 2:**
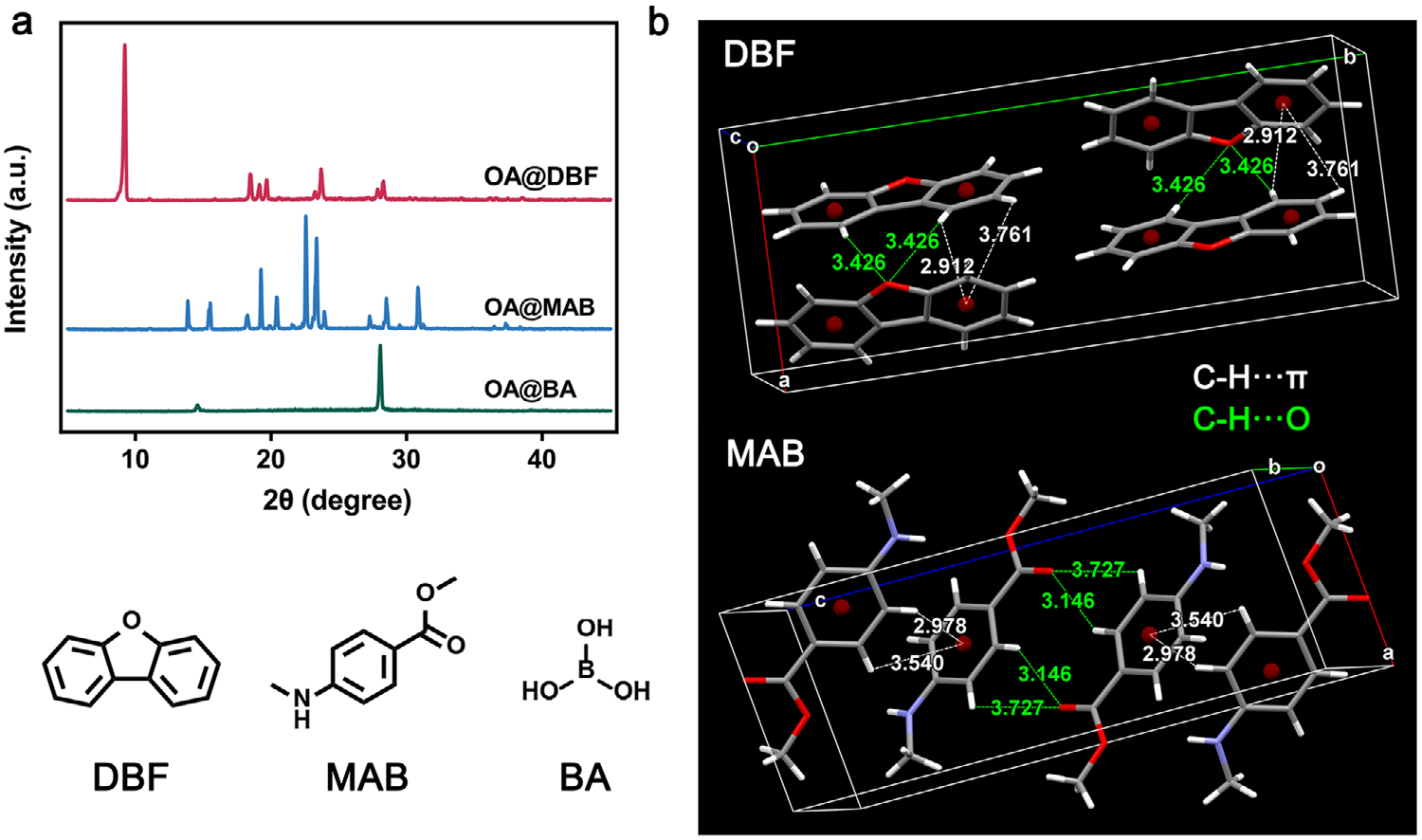
(a) The XRD spectra of OA@DBF, OA@MAB and OA@BA. The molecular formulas of DBF, MAB, and BA. (b) The crystal analysis data of OA@DBF and OA@MAB (m/m = 1:100, only single‐crystal data were resolved. The red, green, and blue lines represent the a‐axis, b‐axis, and c‐axis of the unit cell, respectively).

The crystalline structures of this series of doped materials are characterized by powder X‐ray diffraction (XRD) spectra (Figure 2a). Notably, OA@MAB exhibited sharp and numerous diffraction peaks, indicating a highly ordered crystalline structure of the doped material. OA and MAB (m/m = 1:100) were dissolved in a solution composed of dichloromethane (DCM) with good solubility of the mixture and hexane with poor solubility of the mixture (Experimental Section 4.5), and block‐like crystals were obtained after natural evaporation. As shown in Figure 2b and Table S1, we could resolve the crystal structure of the host MAB from OA@MAB at room temperature. MAB had a monoclinic crystal system and exhibited strong hydrogen bonding interactions. Within one crystallographic unit cell, the distance between H‐O groups was approximately 3.1 Å, indicating an effective hydrogen bond. Hydrogen bonds and C─H···π interactions dominated the crystal packing, with a centroid‐H distance of around 3.0 Å. Van der Waals interactions among molecules arranged along the a‐ and c‐axes further stabilized the three‐dimensional crystal structure [13]. The crystal growth and structure resolution of OA in the DBF matrix turned out to be more demanding, and the crystallization quality was inferior to that of OA@MAB. Here, block‐like crystals of OA@DBF (m/m = 1:100) were prepared by natural evaporation from an ethanol solution, and the structure of the host DBF was resolved at low temperature. DBF had an orthorhombic crystal system, stacking mainly via C─H⋯O interaction between the H─O groups (∼3.4 Å), facilitating the formation of aromatic layers along the b‐axis. The distance between an aromatic ring center to hydrogen was 2.9 Å and 3.8 Å, basically within the typical range of C─H···π interaction (2.5–3.5 Å) [14]. This verifies the existence of such a weak intermolecular force in DBF. Nevertheless, these interactions were weaker than those in the MAB matrix, accounting for the inferior crystallinity of OA@DBF compared to OA@MAB [15]. Moreover, the XRD diffraction peaks of OA@DBF and OA@BA were relatively weaker; this is plausibly due to the interactions occurring when forming composites, which affected the structures to some extent. All OA@DBF, OA@MAB and OA@BA had distinct diffraction peaks, confirming that they were crystalline materials.

We also characterized the micro‐morphologies of OA@DBF, OA@MAB, and OA@BA. As shown in Figure S13, OA@DBF exhibited irregular block‐like and rod‐like shapes without any obvious pattern or orientation, presenting a relatively loose and disordered packing state. The surface of OA@MAB featured distinct striated structures, with some scattered small particles attached to or filling the spaces between these striations, exhibiting a certain hierarchical structure and a higher degree of order compared to OA@DBF. OA@BA was more uniform, displaying a relatively regular stacking arrangement, with a significantly higher degree of order than the former two.

**FIGURE 3:**
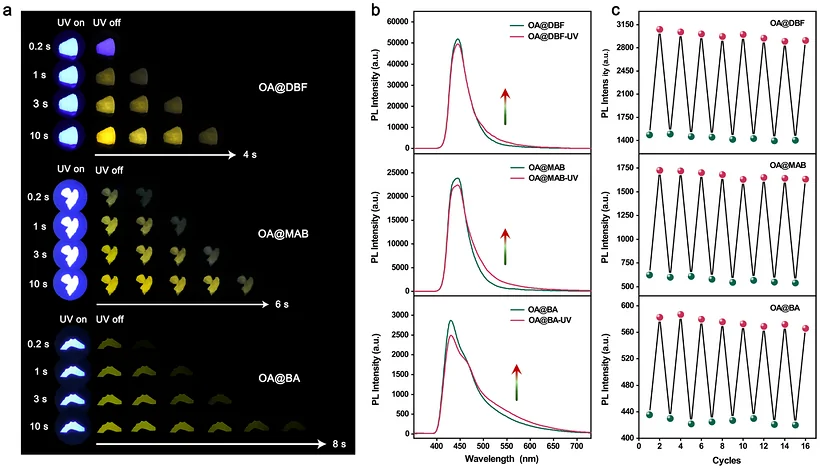
(a) Time‐dependent afterglow photographs of OA@DBF, OA@MAB and OA@BA. (b) The PL spectra before and after UV irradiation of OA@DBF, OA@MAB and OA@BA. (c) The PL intensity at 550 nm before and after alternating UV irradiation of OA@DBF, OA@MAB and OA@BA.

As expected, OA@DBF, OA@MAB and OA@BA all exhibited photoactivated yellow RTP characteristics (Figure 3a). With continuous UV irradiation, OA@DBF, OA@MAB and OA@BA displayed longer‐lasting and brighter yellow afterglow. Among them, OA@BA exhibited the longest afterglow duration, which may be attributed to the inherent advantages of BA as a host matrix sustaining a developed rigid hydrogen bond network, which effectively reduced non‐radiative decay caused by thermal vibrations [16]. Figure 3b shows the changes in PL spectra of OA@DBF, OA@MAB, and OA@BA before and after photoactivation (with a 400 nm filter). The phosphorescent emission peak at 550 nm gradually increased for all three doped materials, while the emission peak in the lower‐wavelength region showed a decreasing trend. This may be attributed to photodamage of the materials under continuous UV irradiation. Figure 3c characterizes the reversibility of OA@DBF, OA@MAB, and OA@BA under photoactivation. After multiple cycling tests, all three doped materials exhibited good activation and recovery capabilities under repeated UV irradiation, indicating that their photoactivation properties are relatively stable and that they possess good potential for practical applications.

It was worth noting that only OA@DBF exhibited blue‐violet afterglow under brief irradiation, whereas OA@MAB and OA@BA only displayed yellow afterglow. Based on the temperature‐dependent measurements (Figure S14 and Table S2), the delayed emission peak intensity of OA@DBF at 335 nm and 400 nm increased with rising temperature, and its lifetime first increased and was followed by a decrease. Together with the fluorescent and phosphorescent spectra of OA, DBF, and OA@DBF at room temperature (Figures 1b and S15), it could be inferred that these two emission peaks originated from the TADF processes of the DBF and OA, respectively. The delayed emission peak at 550 nm decreased with increasing temperature, and the lifetime showed the same trend, corresponding to phosphorescence from OA.

Then we tested the temperature‐dependent delayed emission spectra and lifetime curves of OA@MAB and OA@BA. As shown in Figure S16 and Table S3, delayed emission peaks of OA@MAB were mainly located at 420 nm and 500 nm at 77 K. while the temperature increased, the intensity of both emission peaks showed a decreasing trend. The luminescent lifetime at 420 nm and 550 nm both decreased with increasing temperature, similar to the long‐wavelength emission peak of OA@DBF, which is assigned to phosphorescence from OA. As shown in Figure S17 and Table S4, the temperature‐dependent changes in the delayed emission peaks of OA@BA were like those of OA@MAB. The emission peak intensity at 550 nm decreased with increasing temperature, and the lifetime also decreased, which is attributed to the guest OA. Interestingly, at a high temperature of 427 K, OA@BA still exhibited strong phosphorescent emission with a lifetime of 218.80 ms, indicating that this system has potential as a high‐temperature phosphorescent material. The property might be attributed to the thermal dehydration of BA at high temperature, leading to the formation of boron oxides and the generation of oxygen holes in the matrix. These oxygen holes can more effectively capture and stabilize electrons, promoting electron‐hole recombination within the doped system and ultimately enabling phosphorescent emission at high temperature [17].

Subsequently, we studied the excitation‐dependent delayed emission of OA@DBF, OA@MAB, and OA@BA. As shown in Figure S18, the emission wavelengths of OA@DBF and OA@MAB were concentrated, and their emission peaks remained unchanged under different excitation wavelengths. The change in excitation wavelength did not significantly alter the position of the main emission peak, suggesting that the luminescence originates from only one chromophore and there is no excitation‐dependent behavior. However, the phosphorescent intensity and emission wavelength ranges of OA@BA changed significantly with varying excitation wavelengths. This might be attributed to the thermal dehydration of BA at high temperature, which generates multiple emission centers and complex energy levels. Different excitation wavelength could select different energy level transitions, giving rise to broad phosphorescence and fluctuations in intensity [18].

According to Figures 2a and S19, the XRD spectra of the materials before and after doping remained essentially unchanged, with the characteristic diffraction peaks of the matrices preserved. Characteristic diffraction peaks related to the guest OA were absent, and we did not observe peak shifts or the formation of a new phase. This indicates that the introduction of the guest OA (1 wt%) does not significantly affect the crystal structure of the host materials, and the host crystalline phase remains intact.

To better understand the luminescent mechanisms, first, we cultivated needle‐like single crystals of OA at room temperature (DCM/hexane = 1:1, v/v). OA consists of a furan ring fused with two naphthalene units. This fusion extends the molecular conjugation pathway, forming a large π‐electron delocalization domain [19]. As presented in Figure S20 and Table S5, the benzene rings in the single crystal structure of OA tend to be coplanar. Benefiting from abundant intermolecular weak interactions, including C─H⋯π and C─H⋯O contacts in the crystal packing, the conjugated planar structure facilitates intramolecular electron delocalization and reduces excited‐state energy dissipation, thereby enhancing the fluorescent emission efficiency. This conclusion to some extent explains the high PLQY (80%) of OA powder at room temperature. Furthermore, the rigidity of the crystal reduces energy loss induced by molecular vibration and rotation, increasing the probability of radiative transitions, and improves both luminescent intensity and stability.

The UV–vis absorption spectra of the OA in implicit THF solution treated using polarizable continuum model (PCM) are shown in Figure S21a, along with the simulations of electronic and vibronic progression for S_0_ → S_1_ transition. The experimental spectra exhibited well‐resolved fine structure, characteristic of rigid conjugated systems and in good agreement with time‐dependent density functional theory (TDDFT) calculations. The 0‐0 electronic transition represented the main absorption band, whereas the 0‐n (n = 1, 2, 3) vibronic transitions contributed to the high‐energy tail of absorption, all together reproducing the broad and asymmetric experimental absorption profile. For the emission spectra shown in Figure S21b, the experimental spectrum consisted of a 0‐0 electronic transition at 390 nm and a progression of 0‐n (n = 1, 2, 3) vibronic transitions arising from the same vibrational mode, while higher‐order progressions contributed less.

**FIGURE 4:**
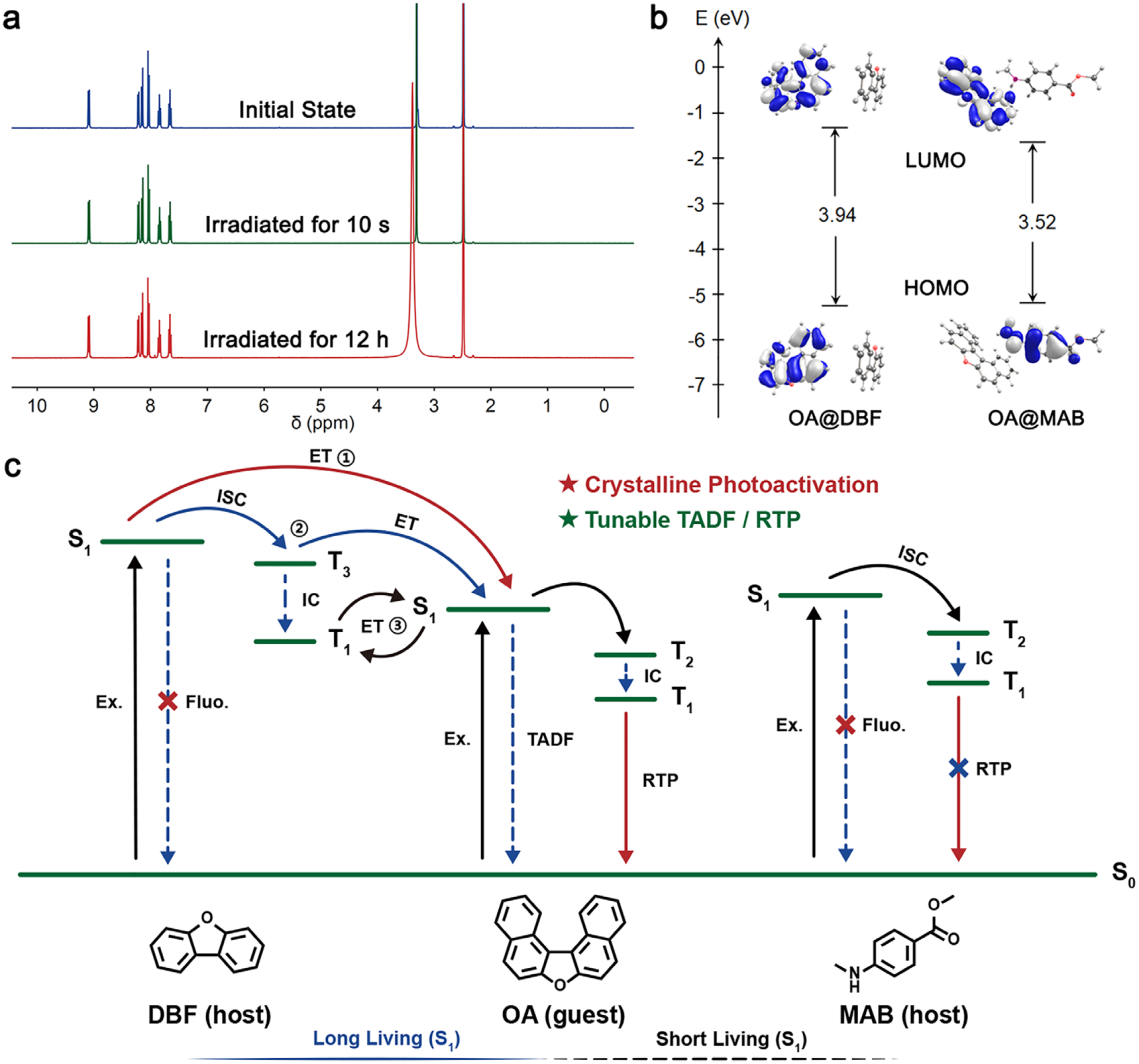
(a) ^1^H NMR (400 MHz, DMSO‐*d*_6_) spectra of OA before and after UV irradiation for 10 s and 12 h. (b) The optimal molecular geometries and HOMO/LUMO analysis of OA@DBF and OA@MAB in the S_0_ state. (c) The schematic diagram of luminescent mechanisms.

Since OA displayed unique photoactivation behavior, it has been documented [20] that certain molecules are susceptible to UV irradiation, which can induce structural degradation and even configurational alterations. Therefore, OA was dissolved in DMSO‐*d*_6_, and ^1^H NMR spectra were recorded before and after 10 s and 12 h of continuous irradiation (Figure 4a). In both cases, the ^1^H NMR chemical shifts, peak shapes, and integration ratios matched those of the original unirradiated OA. No new proton signals were observed, and all original peaks remained unchanged (neither disappearing nor shifting). These results indicate that UV irradiation does not induce any molecular structural changes in OA. Figure S22a presents the absorption changes of OA@2‐MeTHF before and after UV irradiation at 77 K, while Figure S22b–d characterize the absorption changes of OA@DBF, OA@MAB, and OA@BA at room temperature. Specifically, at low temperature, the absorption of OA@2‐MeTHF increased overall upon UV irradiation, particularly in the visible region above 400 nm. Notably, neither the peak positions nor the peak shapes of the OA@2‐MeTHF absorption spectra changed during this case, and no new characteristic absorption peaks appeared. Meanwhile, the absorption curves of OA@DBF, OA@MAB, and OA@BA were almost identical before and after UV irradiation. This result further supports the fact that the molecular structure of OA remains unchanged under UV irradiation.

Upon excitation, UV light first excites the molecule from the ground state to the higher singlet excited state, generating prompt fluorescence after the fast S_n_ → S_1_ non‐radiative quenching. With continued irradiation, the molecule might undergo a transition to a high‐spin state via intersystem crossing (ISC), converting from the singlet to the triplet manifold. For an isolated OA molecule at room temperature, triplet state energy rapidly dissipates, making phosphorescence unobservable. Doping can often improve the situation by suppressing non‐radiative collisional quenching of phosphorescence through a rigid environment, host‐assisted energy transfer (ET), and enhanced ISC, thereby inducing phosphorescent emission. We first calculated the highest occupied molecular orbital (HOMO) and the lowest unoccupied molecular orbital (LUMO) of the two complexes (OA@DBF and OA@MAB) at S_0_ state as shown in Figure 4b. Since the HOMO/LUMO distribution and energies (3.94 eV) in the complex OA@DBF are identical to those of isolated OA (Figure S23 and Table S6), the interaction between DBF and OA is considered to be weak, primarily providing physical isolation of OA and a rigid environment by DBF rather than significant electronic coupling, allowing these two molecules to be calculated separately for future analysis of excited states in co‐assembly systems. The luminescent mechanisms are shown in Figure 4c, and the excited state calculations are summarized in Table 1. Based on the analysis of energy level differences and transition probabilities, we speculate that there are three main pathways that could be involved in TADF stimulation of OA@DBF (labelled 1, 2 and 3 in the diagram). Among them, pathway 1 corresponds to ET from the S_1_ state of DBF to the S_1_ state of OA, followed by radiative decay of OA from S_1_ to S_0_, which directly generates TADF.

**TABLE 1:** Calculated vertical excitation wavelength (λ_vert_) (experimental values (λ_exp_) in parentheses), adiabatic energy (E_ad_), oscillator strength (f), orbital assignment, and configuration interaction (CI) coefficient of S_0_ → S_1_ and S_0_ → T_n_ transitions of OA, DBF, and MAB using TDDFT (TDA) calculations.

compound	excited state	λ_vert_ (λ_exp_) (nm)	E_ad_ (eV)	f	assignment	CI coefficient
OA	T_2_	445	2.96	—	H – 2 → L	0.21624
					H – 1 → L	−0.29466
					H → L + 1	0.57592
	T_1_	604 (‐)	2.34	—	H → L	−0.68560
	S_1_	360 (405)	3.59	0.57	H → L	0.68153
DBF	T_3_	306	4.19	—	H – 2 → L	−0.31513
					H – 2 → L + 2	0.17225
					H – 1 → L	−0.34134
					H → L + 1	−0.49381
	T_2_	356	3.66	—	H – 2 → L + 1	−0.15998
					H → L	−0.66541
	T_1_	445	3.13	—	H – 3 → L + 3	−0.10086
					H → L	−0.68747
	S_1_	283	4.49	0.032	H – 1 → L	−0.62838
					H → L + 1	−0.29723
MAB	T_2_	348	3.25	—	H → L	−0.10305
					H → L + 1	−0.69201
	T_1_	432	2.57	—	H → L	0.69521
	S_1_	431	3.77	<10^−4^	H – 1 → L	0.10657
					H → L	0.68888

For pathways 2 and 3, the process begins with ISC from S_1_ to T_3_ in DBF, generating a triplet exciton stored on DBF. From this state, two distinct routes are possible: in pathway 2, it directly transfers to OA(S_1_) via ET; in pathway 3, it first undergoes internal conversion (IC) from T_3_ to T_1_ within DBF, followed by ET to OA (S_1_). Both routes finally generate TADF upon ET to OA(S_1_). However, the ET in pathway 3 is thermodynamically prohibited (T_1_ of DBF is 0.46 eV lower than S_1_ of OA) and requires significant thermal activation, which may reduce the overall TADF efficiency by promoting exciton loss through back‐transfer and non‐radiative decay. It should be noted that the oscillator strength (f = 0.0320) of S_1_ → S_0_ fluorescence by DBF is very weak, and the total S_1_ → T_n_ (n = 1, 2, 3) ISC rate constant is relatively small (the relevant spin‐orbit coupling (SOC) matrix elements are shown in Table 2; Table S7). These factors collectively result in a low ISC probability of DBF, thereby limiting the efficiency of pathways 2 and 3. In contrast, the HOMO/LUMO distribution in the OA@MAB complex shows a significant difference: the HOMO is mainly localized on MAB, while the LUMO is predominantly distributed on OA. Furthermore, as shown in Figure S24, HOMO – 1 is localized on OA, resulting in an HOMO – 1/LUMO gap of OA@MAB equal to 3.94 eV, which is identical to the isolated OA bandgap. This means weak electronic coupling between OA and MAB, similar to OA@DBF. MAB itself exhibits strong ISC capability from S_1_ to T_1_ and T_2_ states, while the oscillator strength (f < 10^−4^) for S_1_ → S_0_ fluorescence is even smaller than that of DBF, making MAB itself less prone to emit fluorescence. However, precisely because the ISC of MAB is too strong, the excited‐state energy is rapidly transferred to the triplet manifold, which hinders any ET to OA. As a result, the OA@MAB complex system cannot exhibit TADF. Experimental results showed that MAB did not exhibit intrinsic RTP when serving as a host. Notably, it has been reported that MAB can emit long‐lifetime blue RTP when used as a guest dopant [21]. Therefore, we speculate that when MAB acted as a host, it tends to aggregate, leading to aggregation‐caused quenching (ACQ) of excited‐state energy, further suppressing phosphorescent emission. Experimental and theoretical calculations collectively indicate that the observed yellow phosphorescence originates from the OA itself. While DBF, MAB, and BA exhibit varied intrinsic properties as hosts, they all effectively enhance OA's phosphorescence by increasing rigidity and suppressing non‐radiative transitions through physical environmental regulation. Notably, MAB exhibits stronger crystallinity than DBF (Figure 2b and Table S1). While the lower photoactivation efficiency results in weaker RTP under low‐energy in situ photoactivation, its highly rigid crystalline environment more effectively stabilizes the photoactivated triplet states, thereby enabling a greater enhancement of RTP under continuous irradiation.

**TABLE 2:** SOC matrix elements and ISC rate constants (S^−1^) of OA, DBF and MAB at S_1_ geometry using ADF spin‐orbit calculations.

compound	⟨φS_1_|H_SO_|φT_1_⟩	⟨φS_1_|H_SO_|φT_2_⟩	⟨φS_1_|H_SO_|φT_3_⟩	k_ISC_ (S_1_‐T_1_)	k_ISC_ (S_1_‐T_2_)	k_ISC_ (S_1_‐T_3_)	k_ISC_ (S_1_‐T_n_)
OA	0.02	0.11	—	1.83 × 10^0^	5.43 × 10^5^	—	5.43 × 10^5^
DBF	0.02	0	0.05	3.31 × 10^0^	0	7.83 × 10^6^	7.83 × 10^6^
MAB	7.71	33.45	—	2.44 × 10^10^	2.50 × 10^12^	—	2.52 × 10^12^

As given by the Clausius‐Clapeyron equation [22], the vapor pressure of liquid oxygen at 77 K is extremely low (∼10^−6^–10^−7^ atm), which indicates that the partial pressure of gaseous oxygen can be neglected at that temperature. It could be considered oxygen‑free under that condition. Since the photoactivation phenomenon still occurred in OA@2‐MeTHF at 77 K, suggesting that oxygen does not play a dominant role in this system and T_1_ state of OA is long‐lived. Consequently, there is a need for a threshold accumulation of emissive T_1_ exciton density before phosphorescence can be observed with the naked eye. UV light acts as a control mechanism for photophysical processes, including excited‐state carrier density and transition probability. There is no photochemical decomposition or structural isomerization occurring, which means the intrinsic electronic states and molecular structure of the material remain unchanged.

**FIGURE 5:**
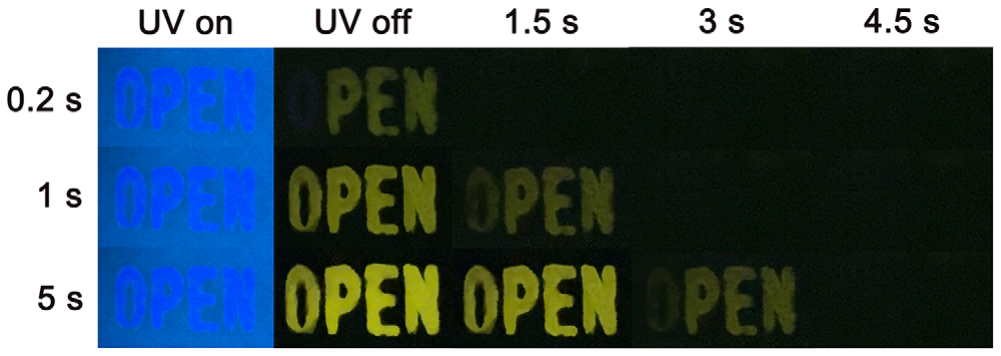
Time‐dependent anti‐counterfeiting label “OPEN”.

By using these yellow afterglow‐doped materials together, different anti‐counterfeiting effects showed up across various time scales. We fabricated an anti‐counterfeiting label that read “OPEN”, and the detailed preparation steps are provided in Experimental Section 4.7. As shown in Figure 5, the stamp for “O” was dipped into “Ink 1”, and the stamp for “PEN” was dipped into “Ink 2”. Then we pressed the stamps onto filter paper, let it air‐dry, and obtained the “OPEN” anti‐counterfeiting label. When irradiated with UV light for 0.2 s and then turned off, the label on the filter paper exhibited a weak yellow afterglow for “PEN” lasting 1 s, while “O” showed a light purple afterglow that was even weaker than that of “PEN”. After irradiation with UV light for 1 s and then turned off, the whole “OPEN” label emitted a yellow afterglow lasting 2 s, with the “O” part remaining relatively weaker. After 5 s of UV exposure followed by turning off the UV light, the label displayed a bright yellow afterglow for “OPEN”. In that case, the afterglow of “O” held for 3 s and stayed weaker, while “PEN” kept its afterglow for almost 4 s. Throughout the entire dynamic process, the label “OPEN” gradually turned into “PEN” after 2 s.

## 3 Conclusion

In summary, we demonstrate for the first time that photoactivated yellow RTP and TADF can be regulated in crystalline materials without any pretreatment by doping OA into DBF, MAB, and BA matrices. Host‐guest energy level matching, exciton lifetime, molecular packing, and crystallinity collectively govern the RTP and TADF emission pathways. Mechanistic investigations suggest that photoactivation in these crystalline systems originates from increased excited‐state carrier concentrations and dynamically optimized transition probabilities under UV irradiation. These findings provide insights into photoactivated luminescence in crystalline systems and offer design principles for developing tunable afterglow materials for time‐dependent anti‐counterfeiting and information encryption.

## 4 Experimental Section

### 4.1 Materials

Dinaphthalen[2,1‐b:1′,2′‐d]furan (OA, 98%) and dibenzofuran (DBF, 98%) were purchased from TCI (Shanghai). Methyl 4‐(methylamino)benzoate (MAB, 98%) and boric acid (BA, 99.99%) were purchased from Bidepharm. The above materials were purified prior to use.

### 4.2 Equipment

Absolute photoluminescent (PL) quantum yields (PLQY) were obtained on a spectrometer C11347‐11 (Hamamatsu, Japan). ^1^H and ^13^C NMR spectra were obtained on a Bruker AV‐400 spectrometer (Bruker, Germany). High‐resolution electron impact (EI) mass spectra were obtained on an EI‐TOFMS (Waters, USA). UV–vis absorption spectra were obtained on a Cary 60 spectrophotometer (Agilent Technologies, USA). PL spectra were obtained on a FluoroMax‐4 spectrometer (HORIBA, Japan). Fluorescent, phosphorescent (phosphorescent mode; delay time = 0.1 ms; gate time = 2.0 ms), and lifetime of delayed emission spectra were obtained on a Cary Eclipse spectrophotometer (Agilent Technologies, USA). X‐ray diffraction (XRD) spectra were obtained on a SmartLab (Rigaku, Japan). Scanning electron microscopy (SEM) images were obtained on a S‐3400N (Hitachi, Japan). Absorption spectra before and after UV irradiation were obtained on a QE Pro high‐sensitivity spectrometer (Ocean Insight, USA). Unless otherwise specified, spectral data were obtained in‐situ under UV irradiation. Unless otherwise specified, the equipment and parameters described above were used throughout this work.

### 4.3 Purification of the Starting Materials

OA: Purify three times by silica gel column chromatography (PE) to obtain a white powder.

^1^H NMR (400 MHz, DMSO‐*d*_6_) δ 9.09 (d, *J* = 8.5 Hz, 2H), 8.22 (dd, *J* = 8.1, 1.4 Hz, 2H), 8.15 (d, *J* = 8.9 Hz, 2H), 8.04 (d, *J* = 8.9 Hz, 2H), 7.85 (m, 2H), 7.66 (dd, *J* = 8.1, 6.8 Hz, 2H). ^13^C NMR (101 MHz, DMSO‐*d*_6_) δ 153.72, 130.92, 129.67, 128.84, 127.71, 126.78, 124.99, 124.71, 118.33, 112.76. HR‐EI‐MS: C_20_H_12_O [M], calculated 268.0888, found 268.0890.

DBF: Purify three times by silica gel column chromatography (PE) to obtain a white powder.

^1^H NMR (400 MHz, DMSO‐*d*_6_) δ 8.16–8.12 (m, 2H), 7.69 (dt, *J* = 8.2, 0.8 Hz, 2H), 7.52 (m, 2H), 7.40 (td, *J* = 7.5, 1.0 Hz, 2H). ^13^C NMR (101 MHz, DMSO‐*d*_6_) δ 155.39, 127.50, 123.50, 123.00, 121.07, 111.58. HR‐EI‐MS: C_20_H_12_O [M], calculated 168.0575, found 168.0577.

MAB: Purify three times by silica gel column chromatography (PE/EA = 4:1, v/v) to obtain a white powder.

^1^H NMR (400 MHz, DMSO‐*d*_6_) δ 7.71–7.66 (m, 2H), 6.57–6.50 (m, 3H), 3.73 (s, 3H), 2.71 (d, *J* = 5.0 Hz, 3H). ^13^C NMR (101 MHz, DMSO‐*d*_6_) δ 166.39, 153.67, 130.94, 115.57, 110.55, 51.12, 29.10. HR‐EI‐MS: C_9_H_11_NO_2_ [M], calculated 165.0790, found 165.0793.

BA: Recrystallized three times with water to obtain a white powder.

### 4.4 Preparation of OA@X

OA@DBF (1 wt%): OA (10 mg) and DBF (1000 mg) were mixed uniformly under ultrasonic agitation and heated to 100°C to form a molten mixture. The molten mixture was then cooled to room temperature to obtain a solidified sample, denoted as OA@DBF.

OA@MAB (1 wt%): OA (10 mg) and MAB (1000 mg) were mixed uniformly under ultrasonic agitation and heated to 100 °C to form a molten mixture. The molten mixture was then cooled to room temperature to obtain a solidified sample, denoted as OA@MAB.

OA@BA (1 wt%): OA (10 mg) and BA (1000 mg) were mixed uniformly under ultrasonic agitation and heated to 200 °C to form a molten mixture. The molten mixture was then cooled to room temperature to obtain a solidified sample, denoted as OA@BA.

### 4.5 Preparation of Crystals

OA@DBF (1 wt%): OA (10 mg) and DBF (1000 mg) were dissolved in ethanol. Block‐shaped crystals were obtained by slow evaporation of the solution under ambient conditions. Crystal structure analysis at low temperature showed that the obtained crystal consisted solely of DBF, with no guest OA present in the crystal lattice.

OA@MAB (1 wt%): OA (10 mg) and MAB (1000 mg) were dissolved in a DCM/hexane mixed solvent (1:1, v/v). DCM and hexane were selected as good and poor solvents for the mixture, respectively. Block‐shaped crystals were obtained by slow evaporation of the solvent mixture under ambient conditions. Crystal structure analysis showed that the obtained crystal consisted solely of MAB, with no guest OA present in the crystal lattice.

OA: OA (1000 mg) was dissolved in a DCM/hexane mixed solvent (1:1, v/v), and needle‐like single crystals were obtained by slow evaporation of the solvent mixture under ambient conditions.

### 4.6 Computational Details

The ground singlet state (S_0_) structures of compounds OA, DBF, and BA were optimized by B3 [23] LYP [24] /6‐31G(d) [25] method within the Gaussian16 software [26]. Simulations with implicit solvent were done with the polarizable continuum model (PCM) [27]. Excited‐state properties were calculated by the time‐dependent density functional theory (TDDFT) [28] method within the Tamm‐Dancoff approximation (TDA) [29], using the same basis set and functional as for the optimization, followed by frequency checks. Spin‐orbit coupling (SOC) matrix elements were obtained by using the Amsterdam density functional (ADF) [30]. The B3LYP hybrid exchange‐correlation functional together with the triple‐zeta polarized (TZP) [31] basis set was employed. The rate constants of ISC [32, 33] were estimated from Equation (1):

(1) $$k_{s_{1}\;\to \;T_{n}}=10^{10}{\langle \varphi_{S_{1}}\left|H_{SO}\right|\varphi_{T_{n}}\rangle }^{2}\cdot F_{om}$$

where E(T_n_) < E(S_1_), $\left\langle \varphi_{S_{1}}\left|H_{SO}\right|\varphi_{T_{n}}\right\rangle$ is the SOC matrix element; F_0m_ is the Franck‐Condon factor, which is approximated through Equation (2):

(2) $$F_{0m}=\sum_{n}\prod_{v}\frac{e^{-y}y^{n_{v}}}{n_{v}!}$$

where $n_{v}=\frac{E_{S_{1}\to T_{n}}}{\omega_{v}}$, the Huang‐Rhys factor γ is assumed to be equal to 0.3 and only one average promotive mode on ω_*v*_ ∼ 1400 cm^−1^ is used.

### 4.7 Preparation of Anti‐Counterfeiting Label “OPEN”

OA (10 mg) and DBF (1000 mg) were dissolved in 10 mL of DCM and stirred thoroughly to obtain “Ink 1”. OA (10 mg) and MAB (1000 mg) were dissolved in 10 mL of DCM and stirred thoroughly to obtain “Ink 2”. The stamp for “O” was dipped into “Ink 1”, while the stamp for “PEN” was dipped into “Ink 2”. The corresponding patterns were then stamped onto filter paper and allowed to dry under ambient conditions.

### 4.8 Statistical Analysis

Figure S21a was normalized using the built‐in normalization function in Origin 2021. The delayed emission lifetimes were fitted by nonlinear least‐squares analysis using the built‐in bi‐exponential decay function (ExpDec2) in Origin 2021.

## Author Contributions

**Lisha Zhang**: conceptualization, methodology, writing – original draft, visualization, data curation, investigation, validation. **Yongping Wu**: conceptualization, methodology, data curation, investigation. **Ihor Sahalianov**: methodology, formal analysis, writing – review and editing, software. **Lei Zhou**: methodology, data curation, investigation. **Glib Baryshnikov**: conceptualization, methodology, writing – review and editing, funding acquisition, supervision, project administration, resources. **Xiang Ma**: conceptualization, writing – review and editing, funding acquisition, supervision, methodology, resources, project administration.

## Funding

This work was partially supported by the Vetenskapsrådet (2022‐06725, 2024–05286), European Research Council (101077649), Swedish Government Strategic Research Area in Materials Science on Advanced Functional Materials (SFO‐Mat‐LiU no. 2009‐00971), Stiftelsen Åforsk (25‐316), National Natural Science Foundation of China (22125803, T2488302).

## Conflicts of Interest

The authors declare no conflicts of interest.

## Supporting information




**Supporting File**: advs77468‐sup‐0001‐SuppMat.docx.

## Data Availability

The data that support the findings of this study are available from the corresponding authors upon reasonable request.
