# Supplementary material for: Photoactivation‐Enabled Tunable Room‐Temperature Phosphorescence and Thermally Activated Delayed Fluorescence in Crystalline Materials
Source: Adv Sci (Weinh). 2026 Aug 29:e77468. Online ahead of print. doi: 10.1002/advs.77468 (PMC13525616; doi:10.1002/advs.77468)
Supplement: Supplementary file 1 [file ADVS-9999-e77468-s001.docx]

Supporting Information

Photoactivation-Enabled Tunable Room-Temperature Phosphorescence and Thermally Activated Delayed Fluorescence in Crystalline Materials

Lisha Zhang^+^,^[A, B]^ Yongping Wu^+^,^[A]^ Ihor Sahalianov,^[B]^ Lei Zhou,^[A]^ Glib Baryshnikov*,^[B]^ and Xiang Ma*^[A]^

A. Lisha Zhang, Yongping Wu, Lei Zhou, Xiang Ma

Key Laboratory for Advanced Materials and Feringa Nobel Prize Scientist Joint Research Center, Frontiers Science Center for Materiobiology and Dynamic Chemistry, School of Chemistry and Molecular Engineering, East China University of Science and Technology, Meilong Road 130, Shanghai, 200237 (China)
E-mail: glib.baryshnikov@liu.se

maxiang@ecust.edu.cn

B. Lisha Zhang, Ihor Sahalianov, Glib Baryshnikov

Laboratory of Organic Electronics, Department of Science and Technology, Linköping University, Bredgatan 33, Norrköping, 60174 (Sweden)

**Figure S1.** ^1^H NMR (400 MHz, DMSO-*d*_6_) spectrum of OA.

**Figure S2.** ^13^C NMR (101 MHz, DMSO-*d*_6_) spectrum of OA.

**Figure S3.** High-resolution EI mass spectrum of OA.

**Figure S4.** The PLQY spectrum of OA powder.

**Figure S5.** (a) The absorption spectra of OA in various solvents. (b) The PL spectra of OA in various solvents (c = 3.0 × 10^−5^ mol·L^−1^).

**Figure S6.** ^1^H NMR (400 MHz, DMSO-*d*_6_) spectrum of DBF.

**Figure S7.** ^13^C NMR (101 MHz, DMSO-*d*_6_) spectrum of DBF.

**Figure S8.** High-resolution EI mass spectrum of DBF.

**Figure S9.** ^1^H NMR (400 MHz, DMSO-*d*_6_) spectrum of MAB.

**Figure S10.** ^13^C NMR (101 MHz, DMSO-*d*_6_) spectrum of MAB.

**Figure S11.** High-resolution EI mass spectrum of MAB.

**Figure S12.** The delayed emission lifetime curves of OA@DBF with different doping concentrations.

**Table S1.** Crystal data of OA@MAB and OA@DBF.

| compound | OA@MAB | OA@DBF |
| --- | --- | --- |
| empirical formula | C_9_H_11_NO_2_ | C_12_H_8_O |
| formula weight | 165.19 | 168.18 |
| temperature/K | 298 | 200 |
| crystal system | monoclinic | orthorhombic |
| space group | P2_1_/n | Pnma |
| a/Å | 6.5072(8) | 7.6006(9) |
| b/Å | 7.6109(9) | 19.153(2) |
| c/Å | 17.504(2) | 5.8101(7) |
| α/° | 90 | 90 |
| β/° | 96.555(6) | 90 |
| γ/° | 90 | 90 |
| volume/Å^3^ | 861.21(18) | 845.78(18) |
| Z | 4 | 4 |
| ρ_calc_g/cm^3^ | 1.274 | 1.321 |
| μ/mm^‑1^ | 0.742 | 0.656 |
| F(000) | 352.0 | 352 |
| crystal size/mm^3^ | 0.2 × 0.17 × 0.15 | 0.28 × 0.18 × 0.05 |
| radiation | CuKα (λ = 1.54178) | CuKα (λ = 1.54178) |
| 2θ range for data collection/° | 10.174 to 137.996 | 9.234 to 136.854 |
| index ranges | -7 ≤ h ≤ 7, -9 ≤ k ≤ 8,  -21 ≤ l ≤ 20 | -9 ≤ h ≤ 8, -23 ≤ k ≤ 21,  -7 ≤ l ≤ 6 |
| reflections collected | 12829 | 10252 |
| independent reflections | 1573 [R_int_ = 0.0575, R_sigma_ = 0.0372] | 797 [R_int_ = 0.0594,  R_sigma_ = 0.0332] |
| data/restraints/parameters | 1573/0/112 | 797/0/61 |
| goodness-of-fit on F^2^ | 1.128 | 1.082 |
| final R indexes [I ≥ 2σ (I)] | R_1_ = 0.0407,  wR_2_ = 0.1252 | R_1_ = 0.0634,  wR_2_ = 0.1712 |
| final R indexes [all data] | R_1_ = 0.0427,  wR_2_ = 0.1277 | R_1_ = 0.0654,  wR_2_ = 0.1740 |
| largest diff. peak/hole/eÅ^-3^ | 0.16/-0.12 | 0.59/-0.42 |

**Figure S13.** The SEM images of (a) OA@DBF, (b) OA@MAB and (c) OA@BA.

**Figure S14.** (a) The delayed emission spectra of OA@DBF at different temperature. (b-d) The delayed emission lifetime curves of OA@DBF at different temperature (λ_em_ = 335 nm, 400 nm, 550 nm).

**T****able S2.** The delayed emission lifetime (ms) of OA@DBF at different temperatures.

| λ_em_ (nm) | 77 K | 127 K | 152 K | 177 K | 227 K | 252 K | 277 K | 302 K |
| --- | --- | --- | --- | --- | --- | --- | --- | --- |
| 335 | - | - | - | - | 215.91 | 336.15 | 137.20 | 45.03 |
| 400 | - | - | 12.49 | 94.59 | 383.72 | 403.16 | 184.95 | 65.79 |
| 550 | 1896.63 | 1763.61 | 1670.06 | 1608.91 | 1311.26 | 1146.11 | 753.09 | 528.18 |

**Figure S15.** The fluorescent and phosphorescent spectra of DBF and OA@DBF at room temperature.

**Figure S16.** (a) The delayed emission spectra of OA@MAB at different temperature. (b-c) The delayed emission lifetime curves of OA@MAB at different temperature (λ_em_ = 420 nm, 550 nm).

**Table S3.** The delayed emission lifetime (ms) of OA@MAB different temperatures (under low-energy in-situ conditions, the effective photoactivated state produced was lower than OA@DBF).

| λ_em_ (nm) | 77 K | 127 K | 177 K | 227 K | 277 K | 327 K |
| --- | --- | --- | --- | --- | --- | --- |
| 420 | 25.31 | 3.61 | 2.96 | - | - | - |
| 550 | 727.70 | 612.45 | 532.12 | 507.82 | 386.71 | 107.12 |

**Figure S17.** (a) The delayed emission spectra of OA@BA at different temperature. (b-c) The delayed emission lifetime curves of OA@BA at different temperature (λ_em_ = 550 nm).

**Table S4.** The delayed emission lifetime (ms) of OA@BA at different temperatures.

| λ_em_ (nm) | 77 K | 127 K | 177 K | 227 K | 277 K | 327 K | 377 K | 427 K |
| --- | --- | --- | --- | --- | --- | --- | --- | --- |
| 550 | 725.91 | 668.01 | 660.78 | 647.14 | 640.92 | 632.63 | 475.70 | 218.80 |

**Figure S18.** Excitation-delayed emission spectra of (a) OA@DBF, (b) OA@MAB and (c) OA@BA.

**Figure S19.** The XRD spectra of (a) OA, (b) DBF, (c) MAB, and (d) BA.

**Figure S20.** Interactions of OA crystals (a) Overall, (b) Partial (the red, green and blue lines represent the a-axis, b-axis and c-axis of the unit cell, respectively).

**Table S5.** Crystal data of OA.

| compound | OA |
| --- | --- |
| empirical formula | C_20_H_12_O |
| formula weight | 268.30 |
| temperature/K | 297 |
| crystal system | triclinic |
| space group | P1 |
| a/Å | 9.277(7) |
| b/Å | 15.312(17) |
| c/Å | 37.213(18) |
| α/° | 93.21(3) |
| β/° | 93.75(3) |
| γ/° | 97.65(3) |
| volume/Å^3^ | 5217(7) |
| Z | 14 |
| ρ_calc_g/cm^3^ | 1.196 |
| μ/mm^‑1^ | 0.566 |
| F(000) | 1960.0 |
| crystal size/mm^3^ | 0.52 × 0.08 × 0.02 |
| radiation | CuKα (λ = 1.54178) |
| 2θ range for data collection/° | 2.384 to 136.484 |
| index ranges | -11 ≤ h ≤ 10, -16 ≤ k ≤ 16, -44 ≤ l ≤ 43 |
| reflections collected | 79162 |
| independent reflections | 29773 [R_int_ = 0.0675, R_sigma_ = 0.0728] |
| data/restraints/parameters | 29773/3/2642 |
| goodness-of-fit on F^2^ | 1.015 |
| final R indexes [I ≥ 2σ (I)] | R_1_ = 0.0776,  wR_2_ = 0.2145 |
| final R indexes [all data] | R_1_ = 0.1049,  wR_2_ = 0.2452 |
| largest diff. peak/hole/e Å^-3^ | 0.33/-0.33 |
| flack parameter | 0.53(17) |

**Figure S21.** The experimental and TDA computational (a) absorption, (b) emission spectra of OA in THF. Electronic emission wavelength is 390 nm at S_1_ state.

**Figure S22.** The absorption spectra before and after UV irradiation (a) 77 K, OA@2-MeTHF, (b) room temperature, OA@DBF, (c) room temperature, OA@MAB, (d) room temperature, OA@BA.

**Figure S23.** The optimal molecular geometries and HOMO/LUMO analysis of DBF, MAB and OA at S_0_ state.

**Table S6.** Calculated vertical excitation wavelength (λ_vert_) (experimental values (λ_exp_) in parentheses), adiabatic energy (E_ad_), oscillator strength (f), orbital assignment and configuration interaction (CI) coefficient of S_0_ → S_1_ and S_0_ → T_n_ transitions of OA@DBF and OA@MAB using TDDFT(TDA) calculations.

| compound | excited state | λ_vert_ (λ_exp_) (nm) | E_ad_ (eV) | f | assignment | CI coefficient |
| --- | --- | --- | --- | --- | --- | --- |
| OA@DBF | T_5_ | 352 | 3.71 | - | H - 7 → L | 0.17227 |
|  |  |  |  |  | H - 4 → L + 2 | 0.14357 |
|  |  |  |  |  | H - 1 → L + 2 | 0.52243 |
|  |  |  |  |  | H → L | 0.16180 |
|  |  |  |  |  | H → L + 3 | -0.10792 |
|  |  |  |  |  | H → L + 5 | -0.25551 |
|  |  |  |  |  | H → L + 7 | -0.14157 |
|  | T_4_ | 372 | 3.46 | - | H - 5 → L + 2 | 0.11753 |
|  |  |  |  |  | H -4 → L | 0.20495 |
|  |  |  |  |  | H - 1 → L | 0.65114 |
|  | T_3_ | 446 | 3.13 | - | H - 8 → L + 8 | -0.10036 |
|  |  |  |  |  | H - 1 → L | 0.68739 |
|  | T_2_ | 445 | 2.96 | - | H - 4 → L | -0.21404 |
|  |  |  |  |  | H - 1 → L | -0.29443 |
|  |  |  |  |  | H → L + 2 | 0.57573 |
|  | T_1_ | 604 (550) | 2.34 | - | H → L | 0.68554 |
|  | S_1_ | 361 (397) | 3.58 | 0.59 | H → L | 0.67905 |
| OA@MAB | T_3_ | 447 | 2.96 | - | H - 4 → L | 0.21799 |
|  |  |  |  |  | H - 2 → L | 0.27017 |
|  |  |  |  |  | H - 1 → L + 1 | -0.58570 |
|  | T_2_ | - | - | - | - | - |
|  | T_1_ | 604 (550) | 2.35 | 0 | H -1 → L | 0.68177 |
|  | S_1_ | 536 (402) | 2.65 | 0.0015 | H → L | 0.70459 |

**Table S7.** The energy (eV) of S_1_ and T_n_ of OA, DBF and MAB at S_1_ geometry using ADF spin-orbit calculations.

| compound | E(S_1_) | E(T_1_) | E(T_2_) | E(T_3_) |
| --- | --- | --- | --- | --- |
| OA | 3.38 | 2.27 | 2.90 | 3.39 |
| DBF | 4.28 | 3.26 | 3.55 | 4.16 |
| MAB | 2.92 | 2.61 | 2.75 | 4.10 |

**Figure S24.** The optimal molecular geometries and HOMO - 1/HOMO/LUMO analysis of OA@MAB at S_0_ state.
